# Tricks in All Trades but Ourn

**Published:** 1909-02

**Authors:** Arthur G. Hobbs

**Affiliations:** Atlanta, Ga.


### "TRICKS IN ALL TRADES BUT OURN."*

BY ARTHUR G. HOBBS, M. D., ATLANTA, GA.

The doctor's tricks, in his consultation room or at the patient's bed-side, are for the patient's good and not for the druggist's emoluments. There is a prescription problem, however, that the doctor must solve for his patient and with his druggist. And may we not go still farther? Is it not a fact, a truth, that almost all patent medicines, and many proprietory formulas, are directly or indirectly purloined from some doctor's prescription, sent in good faith to his druggist, to meet a special, and not hypothetical, or imaginary ills.

A prescription is only an instruction to the druggist for the patient's present needs. It is not an order for all future time; and yet the patient, in his ignorance or haste, will vie with the druggist's cupidity to the detriment of the one, and to the small gain of the other.

An order for any other commodity would not be duplicated. But should it be, the loss would only affect the purse, and not the physical well being of the patient.

All doctors write prescriptions, sometimes, that are only placebos when they find nothing else indicated; what intelligent doctor could do otherwise? And shall I say, "by the way?" No; because the following interpolation is apropos: Upon this rock—the application of the immemorial place—be of all true doctors—is founded the so-called Christian Science Treatment. This cult assumes that there is nothing potential in therapeutics; nothing sillier, nor that there is any good in surgical assistance; nothing more absurd. Is this delusion altogether mental? "Pity 'tis, and pity 'tis, 'tis not true." But a little "knowledge does often make mad," especially when applied to medicine, the science of all sciences that has made the greatest strides during the last two decades.

But to return to the caption of this paper. The doctor's tricks are altruistic and not egotistic—for the patient's good only. When we make a prescription of potency, with a power for good, or if misused, for evil, we naturally do not always want it refilled, neither for the patient's possible detriment, nor for the drug-

*Prepared for Fulton County Medical Society.

gist's gain, nor yet for the patent medicine vampire's vaunting.

Then, how are we to meet this emergency? Suppose we use an *Evanescent Ink* in the body of the prescription to last for a week only? The doctor's and the patient's name can be written with the ordinary pen, which will hold fast. But when the druggist is called upon to refill No. 4711 he finds a blank. He wonders why, but he has nothing else to do but to see, or 'phone the doctor before he can refill the prescription, and learn whether or not he should.

The druggist is human, like all of us; he wants a dollar's profit, more or less, from the returned prescription; the doctor will never know, and the patient thinks he has the right to demand its refilling.

This question, as to the rights of the doctor's prescription, has not yet been settled as it should and must be for the patient's good.

The foundation of all patent medicines rest right here, and, most all proprietary medicines are so based. As the refilling goes on and on, and like an endless chain, the ℞ is handed down from one to another. The natural suggestion to the druggist is, Why not make the ℞ a patent, or, else turn it into a proprietary formula?

If we should write on our prescriptions: "Not to be refilled," it takes much time, with the average patient, to explain why this sentence was necessary. But, even the patient will probably disregard it, and the druggist will overlook it, when he finds his stipend is at stake.

The ordinary patient's idea is that a doctor's prescription, once delivered, is a fee-simple title to its perpetual use, whether paid for or not. This is without regard to the good or bad results that its refilling might bring about.

Then why should we not use an *evanescent ink* when we write a potent prescription, for the patient's and for our own good? It is easy to do. It will keep us in touch with our patients, and them with us.

I will suggest a crude formular of an Evanescent Ink:

| | | |
|---|---|---|
| ℞—Iodine -- -- -- -- -- -- -- -- -- -- -- | grs. | 5 |
| Iodide Pot. -- -- -- -- -- -- -- -- -- -- | grs. | 5 |
| Mucil Acacia -- -- -- -- -- -- -- -- -- -- | dra. | 2 |
| Aqua add to -- -- -- -- -- -- -- -- -- -- | oz. | 22 |

Mix and use with an ordinary pen on well glazed paper.

But there are many other and probably better evanescent inks to be found at the book stores, and in some drug stores.

Then if "there are tricks in all trades but ourn," may not any true doctor resort to this one for his patient's good?

805-7-9 English-American Biulding.

